# Supplementary material for: Does the threat of COVID-19 modulate automatic imitation?
Source: PLoS One. 2023 Apr 24;18(4):e0284936. doi: 10.1371/journal.pone.0284936 (PMC10124885; doi:10.1371/journal.pone.0284936)
Supplement: S1 Text — (DOCX) [file pone.0284936.s001.docx]

**Instructions**

You are about to attempt a language comprehension test. Please read each one of the short statements, which have been extracted from news headlines over the past 18 months. These statements are incomplete, and it is your task to fill in the missing words to form a comprehensible sentence by selecting the appropriate boxes near the possible words for you to choose from.

For example;

The chimney sits on of the .

*before, top, roof, flowers*

*Answer: The chimney sits on top of the roof*

**Neutral**

1. Trade to be shortly

*deal, announced, reverse, supply*

1. Libraries to “ of a new era’’

*height, leap, enter, dawn*

1. ‘Lane-keeping’ technology for ‘in the ’

*stairs, motorways, cross, works*

1. Marine becoming one of the growing areas of study

*pinnacle, biology, fastest, sharp*

1. Mystery as of birds plummet into .

*hacking, flock, farmland, wheel*

1. Study shows of dam on nearby orangutan .

*impact, strange, traits, population*

1. Experts in awe at dolphin stampede the of Canada

*ghost, off, coast, fog*

1. temperature on is reached

*moderate, highest, record, wild*

1. Rail service temporarily for essential .

*stopped, generator, repairs, cleansing*

1. Artificial grass is most in British .

*popular, gardens, paper, excitement*

1. to rise

*basement, courses, household, bills*

1. Asteroid Earth

*flies, by, via, rain*

1. US Supreme nomination announced

*new, high, court, theatre*

1. space market momentum

*plagiarised, commercial, gathers, append*

1. Petition to Berlin’s ‘cheeky’ boar

*save, fleeting, wild, remote*

1. to consider when to a plant-based diet

*things, falling, devour, switching*

1. village café to .

*oldest, vaporise, close, longest*

1. Augmented reality holograms can medical .

*fix, help, retirees, practitioners*

**Safe**

1. Manufacturers up the of PPE

*strong, ramp, supply, stem*

1. Larger to retain of remote working

*firms, measures, kettle, smoke*

1. ‘Lane-keeping’ technology for ‘in the ’

*stairs, motorways, cross, works*

1. Older technophobes begin to to technology

*age, fix, fly, take*

1. pursuits and gardening on the since lockdown

*smooth, cello, outdoor, rise*

1. Study shows of dam on nearby orangutan .

*impact, strange, traits, population*

1. Experts in awe at dolphin stampede the of Canada

*ghost, off, coast, fog*

1. temperature on is reached

*moderate, highest, record, wild*

1. cycle to be introduced

*more, lanes, road, truck*

1. Homemade masks could just do the .

*cake, paper, face, trick*

1. to rise

*basement, courses, household, bills*

1. Video officially substitutes the .

*conferencing, hospitality, boardroom, bathroom*

1. US Supreme nomination announced

*new, high, court, theatre*

1. space market momentum

*plagiarised, commercial, gathers, append*

1. Demand soars in conferencing .

*console, video, apps, painting*

1. to consider when to a plant-based diet

*things, falling, devour, switching*

1. village café to .

*oldest, vaporise, close, longest*

1. ‘Handwashing is these days’

*more, predicted, fashionable, common*

**Unsafe**

1. Indoor gyms to reopen to the .

*begin, deceased, family, public*

1. workers raise over PPE shortage

*manual, health, issues, smoke*

1. ‘Lane-keeping’ technology for ‘in the ’

*stairs, motorways, cross, works*

1. ‘Handwashing is no than it was before the outbreak’

*more, predicted, fashionable, common*

1. Visits to the increase as lockdown .

*arena, supermarket, modernise, hits*

1. Study shows of dam on nearby orangutan .

*impact, strange, traits, population*

1. Experts in awe at dolphin stampede the of Canada

*ghost, off, coast, fog*

1. temperature on is reached

*moderate, highest, record, wild*

1. Separate can meet each other’s homes

*households, food, inside, airplanes*

1. What for the workplace during the ?

*occurs, next, pandemic, sports*

1. to rise

*basement, courses, household, bills*

1. go to the shops

*magician, thousands, back, delay*

1. US Supreme nomination announced

*new, high, court, theatre*

1. space market momentum

*plagiarised, commercial, gathers, append*

1. book-up as kicks-in

*restaurants, discount, classrooms, tropical*

1. to consider when to a plant-based diet

*things, falling, devour, switching*

1. village café to .

*oldest, vaporise, close, longest*

1. Pubs and restaurants their doors as are lifted

*raised, open, fires, restrictions*

**Answers**

Neutral primes

1. Commercial space market gathers momentum
2. ‘Lane-keeping’ technology for motorways ‘in the works’
3. Things to consider when switching to a plant-based diet
4. Oldest village café to close
5. New US Supreme Court nomination announced
6. Highest temperature on record is reached
7. Experts in awe at dolphin stampede off the coast of Canada
8. Study shows impact of dam on nearby orangutan population
9. Household bills to rise
10. Trade deal to be announced shortly
11. Libraries to enter “dawn of a new era’’
12. Marine biology becoming one of the fastest growing areas of study
13. Mystery as flock of birds plummet into farmland
14. Rail service temporarily stopped for essential repairs
15. Artificial grass is most popular in British gardens
16. Asteroid flies by Earth
17. Petition to save Berlin’s ‘cheeky’ wild boar
18. Augmented reality holograms can help medical practitioners

Risk-averse/Safe primes

1. Video conferencing officially substitutes the boardroom
2. Manufacturers ramp up the supply of PPE
3. Outdoor pursuits and gardening on the rise since lockdown
4. Homemade face masks could just do the trick
5. Older age technophobes begin to take to technology
6. More cycle lanes to be introduced
7. Larger firms to retain measures of remote working
8. ‘Handwashing is more common these days’
9. Demand soars in video conferencing apps

Risk-tolerant/Unsafe primes

1. Thousands go back to the shops
2. Indoor gyms begin to reopen to the public
3. Visits to the supermarket increase as lockdown hits
4. What next for the workplace during the pandemic?
5. ‘Handwashing is no more common than it was before the outbreak’
6. Separate households can meet inside each other’s homes
7. Health workers raise issues over PPE shortage
8. Pubs and restaurants open their doors as restrictions are lifted
9. Restaurants book-up as discount kicks-in
