# Supplementary material for: Does the threat of COVID-19 modulate automatic imitation?
Source: PLoS One. 2023 Apr 24;18(4):e0284936. doi: 10.1371/journal.pone.0284936 (PMC10124885; doi:10.1371/journal.pone.0284936)
Supplement: S2 Text — (DOCX) [file pone.0284936.s002.docx]

A sub-set of individuals (n = 6) were provided with a series of sentences, which they had to evaluate. That is, they rated each of the sentences in terms of their perceived valence/pleasantness (1-9; 1 = unpleasant, 9 = pleasant), arousal (1-9; 1 = calm, 9 = aroused), dominance (1-9; 1 = controlled, 9 = in control) and socialability (1-5; 1 = very anti-social, 5 = very pro-social). The initial ratings of valence/pleasantness, arousal and dominance were adapted from the rating system used for the International Affective Picture System (IAPS; Bradley & Lang, 1994; Lang et al., 1993) (for examples within automatic imitation, see Grecucci, Balaban et al., 2009; Grecucci, Koch et al., 2011) and facilitated by the self-assessment manikin (Figure 1). Meanwhile, the latter rating of socialability was adapted from Leighton et al., 2010.


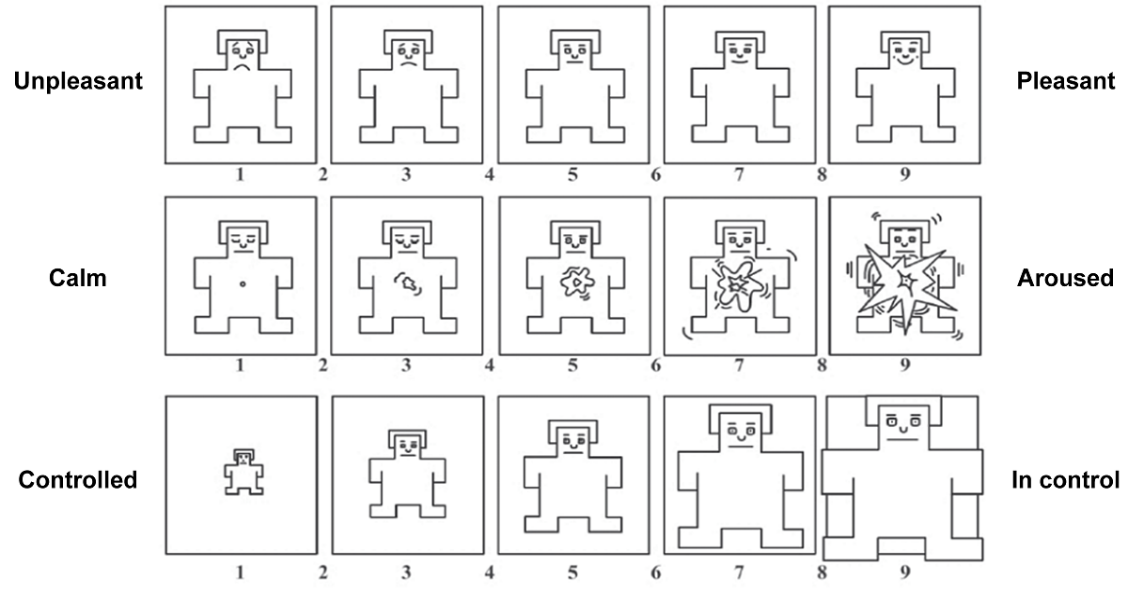


Figure 1. Self-Assessment Manikin (SAM)

There were initially 56 sentences to rate including 28 neutral, 14 safe and 14 unsafe. Based on obtaining relatively similar scores on the abovementioned scale; particularly the rating of socialability, we selected only a portion of these sentences for the experiment. As a result, there were 36 different sentences that were selected including 18 neutral, 9 safe and 9 unsafe.

Of the selected sentences, the participant mean ratings for each scale were statistically analysed using a one-way repeated-measures ANOVA. While there was a significant effect for ratings of valence/pleasantness, *F*(2,10) = 4.73, *p* = .04, *η_p_^2^* = .49, and arousal, *F*(2,10) = 18.10, *p* < .001, *η_p_^2^* = .78, there was no significant effect for ratings of dominance, *F*(2,10) = 3.07, *p* = .09, *η_p_^2^* = .38, and socialability, *F*(2,10) = .09, *p* = .92, *η_p_^2^* = .02 (Table 1).

**Table 1.** Mean participant ratings (±SE) for each of the prime conditions (N.B., safe and unsafe conditions also comprised a portion of the neutral primes)

|  | Neutral | Safe | Unsafe |
| --- | --- | --- | --- |
| Valence/Pleasantness | 4.19 (.22) | 4.80 (.05) | 4.24 (.17) |
| Arousal | 4.30 (.25) | 4.30 (.25) | 5.10 (.29) |
| Dominance | 4.06 (.41) | 4.65 (.41) | 4.24 (.42) |
| Socialability | 2.59 (.22) | 2.64 (.17) | 2.56 (.16) |

**References**

1. Bradley MM, Lang PJ. Measuring emotion: the self-assessment manikin and the semantic differential. J Behav Ther Exp Psy. 1994; 25(1):49-59.
2. Lang PJ, Greenwald MK, Bradley MM, Hamm AO. Looking at pictures: affective, facial, visceral, and behavioral reactions. Psychophysiology. 1993; 30(3):261-73.
3. Grecucci A, Balaban E, Buiatti T, Budai R, Rumiati RI. The emotional control of action: ERP evidence. Arch Ital Biol. 2009; 147(1-2):37-49.
4. Grecucci A, Koch I, Rumiati RI. The role of emotional context in facilitating imitative actions. Acta Psychol. 2011; 138(2):311-5.
5. Leighton J, Bird G, Orsini C, Heyes C. Social attitudes modulate automatic imitation. J Exp Soc Psychol. 2010; 46(6):905-10.
