# Supplementary material for: Does the threat of COVID-19 modulate automatic imitation?
Source: PLoS One. 2023 Apr 24;18(4):e0284936. doi: 10.1371/journal.pone.0284936 (PMC10124885; doi:10.1371/journal.pone.0284936)
Supplement: S3 Text — (DOCX) [file pone.0284936.s003.docx]

We broadly categorised participants’ data collection times as being within the early and late stages of the pandemic. That is, the former was in the midst of widespread transmission and increased hospitalisation including limited access to the vaccine (date range = 22^nd^ Feb, 2021–4^th^ March, 2021) (n = 28), while the latter involved fewer hospital admissions with widespread access to the vaccine across the UK (4^th^ Nov, 2021–18^th^ Feb, 2022) (n = 29).

For the analysis, the fore mentioned timepoints were entered as a between-measures factor into the same factorial design as the main set of analyses from the manuscript. That is, we conducted a three-way mixed-design ANOVA including the between-measures factors Prime (control, safe, unsafe) and Timepoint (earlier, later), and within-measures factor Compatibility (compatible, incompatible).

Focusing on the relevant statistical effects involving the factor of Timepoint, there was no significant main effect of Timepoint, *F*(1,51) = 2.40, *p* = .13, *η_p_^2^* = .05, BF_10_ = .90, nor a significant Prime x Timepoint, *F*(2,51) = .43, *p* = .65, *η_p_^2^* = .02, BF_10_ = .67, Timepoint x Compatibility, *F*(1,51) = .16, *p* = .69, *η_p_^2^* = .00, BF_10_ = .27, and Prime x Timepoint x Compatibility, *F*(2,51) = .07, *p* = .93, *η_p_^2^* = .00, BF_10_ = .21, (see Table 1) interaction.

**Table 1.** Mean participant reaction time (ms) (±SE) within compatible and incompatible stimulus-response trials as a function of data collection timepoints (earlier, later).

|  | Earlier  (Feb, 2021) | | Later  (Nov, 2021 & Feb, 2022) | |
| --- | --- | --- | --- | --- |
|  | Compatible | Incompatible | Compatible | Incompatible |
| Neutral | 479 (±28) | 503 (±28) | 547 (±35) | 553 (±34) |
| Safe | 515 (±21) | 573 (±39) | 525 (±28) | 582 (±49) |
| Unsafe | 508 (±36) | 566 (±45) | 588 (±45) | 641 (±66) |
