# Supplementary material for: Does the threat of COVID-19 modulate automatic imitation?
Source: PLoS One. 2023 Apr 24;18(4):e0284936. doi: 10.1371/journal.pone.0284936 (PMC10124885; doi:10.1371/journal.pone.0284936)
Supplement: Attachment — Submitted filename: Response to Reviewers.docx [file pone.0284936.s004.docx]

**Response to Reviewers**

**Please note, the references to page and line numbers are taken with respect to the manuscript version, ‘Revised Manuscript with Track Changes’*

Editor:

Editor comment/s: As you will see, reviewer 2 is still concerned about the issue of spatial compatibility between the stimulus and the response, which is usually overcome by using orthogonal orientations between the direction of movement and the observer's or imitator's personal body part. It is not proposed that you run control experiments, but it is requested that you explicitly and extensively acknowledge the fact that the effect you are measuring is spurious and contains an un-measurable proportion of simple spatial compatibility rather compared to the genuine action observation effect. This is actually a very big issue in action observation protocols, which should always be controlled for spatial compatibility issues.

*Authors’ response: We fully recognise the potential of spatial compatibility effects contaminating the present findings (Pg. 14, Ln. 319-330). Specifically, we highlight the potential for complex/orthogonal spatial compatibility, where the left-down and right-up stimulus-response mappings may be observed (N.B., only for midline or right hemispace responses).*

Reviewers' comments:

Reviewer #1:

Reviewer comment/s: Thank you for your responses. It is surprising that apriori power analyses weren't conducted. In the participants section you should detail what the current sample size was based on.

*Authors’ response: We would like to thank the reviewer for their constructive comments. The sample size in question was mostly determined by the comparatively limited uptake when recruiting participants combined with the knowledge that the pandemic and national restrictions were nearing an end (Pg. 4-5, Ln. 86-87). That said, the subsequent sample size was consistent with much of the previous literature (Leighton et al., 2010; Wang & Hamilton, 2013) (Pg. 5, Ln. 87-88), while the sensitivity analysis was favourable toward our obtaining an achievable effect size (Pg. 9, Ln. 194-196). In this regard, we consider our sample size sufficient enough for the purposes of the present study.*

Reviewer #2:

Reviewer’s comment/s: On the basis of paradigm description provided in the revised MS I believe that orthogonal-spatial compatibility effects were not considered and the measured effect confounds spatial and imitative influences. Although many existing studies do not take spatial effects into account in their designs (either simple or orthogonal), sufficient evidence on this issue is now available (see papers on automatic imitation paradigm as well as other spatial compatibility tasks). Second, these spatial compatibility effects differ for right- and left-handers. While both subgroups might have had a similar magnitude of compatibility effect in this particular sample, its primary sources can be different for each subgroup. The resulting effect could be due predominantly to orthogonal-spatial compatibility in the right-handers, while the opposite could be true for left-handers. In other words, while inclusion of left-handers ensured larger sample, the measured effects became more confounded. In this context, it is irrelevant that the same stimulus was presented to the entire sample – all responses were confounded to some extent (and this extent is unknown). Given that additional data collection to address this methodological oversight is not feasible at this stage, the issue of orthogonal compatibility has to be reflected in interpretation and discussion of the results because a lack of behavioural modification by social cues can well be due to measurement of spatial, not imitative characteristics. I strongly suggest that authors become familiar with issues associated with automatic imitation paradigm – specifically those related to spatial confounds and external validity of the paradigm (e.g., recently reported lack of association between imitation inhibition using similar paradigm and mimicry).

*Authors’ response:* *We fully recognise the potential of spatial compatibility effects contaminating the present findings (Pg. 14, Ln. 319-323). Specifically, we highlight the potential for complex/orthogonal spatial compatibility, where the left-down and right-up stimulus-response mappings may be observed (N.B., only for midline or right hemispace responses).*

*However, we are unable to reconcile the influence of handedness on such spatial compatibility effects. This is not to say that such an influence does not exist, although we currently fail to comprehend this logic (for response hand effects, see Cho & Proctor, 2004). Along these lines, we may indirectly infer the extent of spatial compatibility within right- and left-handed participants by re-evaluating the compatibility effects as a function of time bin (i.e., slow vs. fast). Here, it would be suggested that compatibility effects primarily unfolding within slow compared to fast reaction time bins would perhaps be more closely associated with automatic imitation (Catmur & Heyes, 2011) (Pg. 14, Ln. 323-327). Meanwhile, compatibility effects that unfold relatively equally across time bins would perhaps earmark spatial compatibility. With this in mind, we found a similarly increased compatibility effect within the slow compared to fast time bins for right- (slow M diff ± SD =53 ms ± 104; fast M diff ± SD = 34 ms ± 59) and left-handed (slow M diff ± SD =50 ms ± 63; fast M diff ± SD = 36 ms ± 59) participants*

Reviewer’s comment/s: Rationale for modest number of trials (i.e., to ensure cooperation of participants) should be provided in the Method section. I disagree with the claim that majority of studies on automatic imitation comprise similar number of trials/participants – large-scale reports are also available.

*Authors’ response: We fully agree that while the currently adopted number of trials is broadly consistent with those adopted within previous studies (e.g., Wang & Hamilton, 2013), it is not consistent with a large amount of other studies surrounding this area of work. Consequently, and as requested, we have provided further detail on our choice of the number of trials (Pg. 7, Ln. 149-152).*

Reviewer’s comment/s: Moreover, individual differences in automatic imitation effect have been revealed and automatic imitation does not manifest consistently at individual level even in neurotypical participants, nor does it have a “standard level” (page 4, line 76).

*Authors’ response: We fully recognise the emerging consensus surrounding the heterogeneity of automatic imitation within the Discussion section (Pg. 14, Ln. 310-318), and alternatively adopt the terminology “typical or baseline” (i.e., within-participant) as opposed to “standard level” (Pg. 4, Ln. 71).*

Reviewer’s comment/s: How did the authors arrive at the reported minimum effect size (ηp2= .043)? Please specify.

*Authors’ response: In line with another reviewer’s request, the minimum effect size to which the test was sensitive was calculated using G*Power. The precise inputs were as follows: Test Family > F Test; Statistical Test > ANOVA – Repeated-measures, within-between; Type of power analysis > Sensitivity; α = .05; 1-β = .80; Total sample = 59; Number of groups = 3; Number of measurements = 2. The relevant parameters for this sensitivity test are stated within the manuscript (Pg. 9, Ln. 194-196).*

Reviewer’s comment/s: It would be useful to report statistical indices including effect size for contrasts in case of a significant interaction term.

*Authors’ response: We are not entirely certain here of the request alluding to effect sizes in addition to the ones currently present for each inferential statistic. Naturally, we welcome further consideration upon potentially receiving another iteration of reviews.*

Reviewer’s comment/s: Sentences on page 15, lines 330-336 belong to the Results section.

*Authors’ response: We fully recognise the principle of describing findings initially within the Results section prior to the Discussion section. However, the findings of interest here are related to an ad hoc observation that pertains to a critical evaluation of the study as it nears its completion. Thus, in order to relocate the description of these particular findings to the Results section, then they would need to be presented prior to forming a rationale in terms of why this analysis was conducted in the first place. Moreover, there are some─all be it rare─instances where similar ad hoc observations are formulated within the Discussion section (e.g., Buckingham et al., 2014; Spengler et al., 2010; Wong et al., 2013). At the same time, it is worthwhile highlighting that the associated inferential statistics are presented elsewhere as a supplement (S3 Text). With this in mind, we have currently elected to retain the description of the findings in question within the Discussion section. Naturally, we welcome the need to revisit this choice if indeed our current explanation is not deemed sufficient.*

Reviewers’ comment/s: In conclusion, my main concern is that it is impossible to tell what was measured in the present study. It is likely that the version of automatic imitation paradigm captures non-social (spatial) rather than social (imitative) compatibility effects. A revision of the MS, where the authors acknowledge this issue and incorporate orthogonal compatibility effect into their interpretations.

*Authors’ response: We fully recognise this possibility within the Discussion section (Pg. 14, Ln. 319-323). Please see previous response to comment/s regarding complex/orthogonal spatial compatibility.*

**References**

Buckingham G, Wong JD, Tang M, Gribble PL, Goodale MA. Observing object lifting errors modulates cortico-spinal excitability and improves object lifting performance. Cortex. 2014; 50:115-24.

Catmur C, Heyes C. Time course analyses confirm independence of imitative and spatial compatibility. J Exp Psychol Hum Percept Perform. 2011; 37(2):409-21.

Cho YS, Proctor RW. Influences of multiple spatial stimulus and response codes on orthogonal stimulus–response compatibility. Percept Psychophys. 2004; 66:1003-17.

Leighton J, Bird G, Orsini C, Heyes C. Social attitudes modulate automatic imitation. J Exp Soc Psychol. 2010; 46(6):905-10.

Spengler S, von Cramon DY, Brass M. Resisting motor mimicry: control of imitation involves processes central to social cognition in patients with frontal and temporo-parietal lesions. Soc Neurosci. 2010; 5(4):401-16.

Wang Y, Hamilton AFC. Understanding the role of the ‘self’ in the social priming of mimicry. PLoS ONE. 2013; 8(4):e60249.

Wong L, Manson GA, Tremblay L, Welsh TN. On the relationship between the execution, perception, and imagination of action. Behav Brain Res. 2013; 257:242-52.
