# Supplementary material for: Does the threat of COVID-19 modulate automatic imitation?
Source: PLoS One. 2023 Apr 24;18(4):e0284936. doi: 10.1371/journal.pone.0284936 (PMC10124885; doi:10.1371/journal.pone.0284936)
Supplement: Attachment — Submitted filename: Response to Reviewers.docx [file pone.0284936.s005.docx]

**Response to Reviewers**

**Please note, the references to page and line numbers are taken with respect to the manuscript version, ‘Revised Manuscript with Track Changes’.*

Editor:

Editor comment/s: As you will see below, the newly included reviewer highlighted relevant points regarding the inclusion of current literature, the rationale for the design as well as other issues with the analyses and reporting thereof. We feel that addressing these points as outlined in their review will strengthen your manuscript.

Reviewer’s comment/s: You will also note that whilst both reviewers provided relevant suggestions on how to address issues of compatibility, their reading of the analysis on both compatibility and time bin interaction differs. I understand that including both factors (compatibility and time-bin interaction) would require a hierarchical analysis approach. To complicate matters further, I have another suggestion by using a different kind of analysis altogether. I would have expected to see a linear mixed-effects model analysis for this type of data/research question. This would have prevented some of the issues identified by the reviewers while at the same time considering other factors (timepoint, gender, differences in priming sentences, individual participant data; and thus more powerful).

*Authors’ response: We are not explicitly clear on the need to adopt a linear mixed effects model within the present study, which otherwise features a fully balanced study design. Most importantly, and as alluded to within the comments above, we potentially risk compromising the requested revisions pertaining to a separate set of reviewer comments, which otherwise continue to endorse a general linear model. With this in mind, we have alternatively attempted to thoroughly acknowledge and/or remedy the above raised issues within the present set of revisions (see below).*

My concern regarding the bin-analysis in its current form is that a higher variance in longer time intervals is simply more likely due to longer time intervals. Please clarify this point. Also, a rationale regarding the fast/slow response mode is missing in the introduction. Was this a post-hoc exploratory analysis? If so, this needs to be stated.

*Authors’ response: We would like to thank the editor for this observation. As a result, we fully recognise the possible confounding influence of variance, where the magnitude of differences could be coincidentally scaled to the magnitude of time; thus, indicating heteroscedasticity. That said, we would perhaps assume that any such influence would render differences between compatible and incompatible trials that are completely random, and not systematic as found within the present study (i.e., compatibility effect-mean reaction time relation would effectively resemble a ‘funnel’ and not necessarily a systematic linear relation).*

*For context, the original logic behind the use of time bins assumed that a compatibility effect related to biologically-specific stimuli, which is synonymous with automatic imitation, would be mostly prevalent within slower time bins. On the other hand, a simple compatibility effect featuring arbitrary, non-biological stimuli would unfold relatively equally across time bins (Catmur & Heyes, 2011). Nevertheless, in the absence of a spatial compatibility control task within the present study, we concede that it is not suitable to positively assert this logic (Pg. 15-16, Ln. 355-357).*

*Of interest, the analysis involving time bins was based on an apriori rationale following previous empirical research findings (Catmur & Heyes, 2011; Cook & Bird, 2011; Heyes & Bird, 2007; Spengler et al., 2010), and thus we have more precisely reported it within the Introduction section including an hypothesis (Pg. 4, Ln. 77-86).*

Therefore, we invite you to submit a revised version of the manuscript that addresses all the points raised during this reviewing cycle. As for the issues surrounding your compatibility analysis, please address all comments and requests for additional data and presentation of findings and clarify the analysis as well as the rationale in the manuscript and/or the response letter.

In addition to the revision, I urge you to include the individual trial RT data in your OSF repository plus participants’ gender (or sex, depending how the participants demographics question). For the data to be of value for certain types of analyses, individual trial data is needed, and gender seems relevant, considering that males have been found to be less Covid-19 risk adverse than females (and you should in fact consider the unbalanced participant cohort as one of your limitations). Finally, I agree with the reviewer for the need of a figure illustrating the stimuli/task in the manuscript and I would recommend uploading your visuals onto the repository.

*Authors’ response: We have now incorporated a spreadsheet on participant characteristics (e.g., sex, handedness) into the original data file, and uploaded individual trial data (see hyperlink, Pg. 8, Ln. 178).*

Reviewer #2:

Reviewer’s comment/s: Although the authors added a brief section on spatial compatibility effects in the revised MS, they support their main interpretation using papers building on different paradigms with potentially different spatial relationships (e.g., participants using both hands). I am therefore unsure if these results can be generalized to the presented study. It has already been revealed that the stimulus hand used here elicits predominantly orthogonal compatibility effects and that purely imitative effects exist, but are rare, more modest in magnitude, and likely elicited by different stimulus. Other stimuli/contexts might not elicit significant compatibility effects at all. This is the reason why reporting strength of the effect (incom-com contrasts) in slow and fast bins is important (compatibility x time bin interaction). Were all incom-com differences in slow and fast bins significant? This could help the interpretation. For example, numerical difference in fast bin between incom and com trials for neutral condition seems very small (12ms) compared to the same difference in unsafe condition (49ms). Moreover, it would be easier on a reader to present these results in a graph rather than table.

*Authors’ response: We have now statistically confirmed the difference in compatibility effects (compatible-incompatible difference) between slow and fast reaction time bins (Pg. 11, Ln. 238). While these differences appeared to vary between groups, this particular statistical effect (i.e., prime x compatibility x time bin) did not reach significance. Along these lines, we have now incorporated a figure (Fig 2; Pg. 11, Ln. 244-246) that more appropriately illustrates the compatibility effects including error bars that indicate the standard deviations, and how there was some degree of overlap between each of the groups in this regard. Please note, we have opted to also retain the table so the reader may observe the original mean values for compatible and incompatible stimulus-response conditions.*

Reviewer #3:

In this manuscript the authors used an automatic imitation task preceded by a sentence comprehension priming task to test for the influence of COVID-related primes on imitation. An imitation effect was observed but no influence of the priming task.

I appreciate that this manuscript has already been reviewed and it’s galling to have to deal with a new set of comments after two rounds of review, so I leave it to the editor’s discretion to decide which of the following points are essential for the authors to address.

Introduction:

Reviewer’s comment/s: The focus on ‘advanced mammals’ in the first sentence seems unnecessary and inaccurate – a wide range of species show forms of imitation, including birds (Richards et al., 2009).

*Authors’ response: We have adapted the opening statement to exclusively focus on imitation within humans, as well as the subsequent citations (Pg. 2, Ln. 15-16).*

Reviewer’s comment/s: The introduction is missing discussion of studies which do not show an effect of social priming on imitation, including several recent and more highly powered studies (Genschow et al., 2022; see also the recent special issue on this topic by Cracco et al., 2022).

*Authors’ response: We have incorporated the suggested citations including the new inclusion of Cracco et al. (2022) (Pg. 2, Ln. 37).*

Reviewer’s comment/s: The rationale for the study doesn’t follow from the discussion of the BIS in the introduction. According to the introduction, the effects of the COVID-related primes should only be seen in participants with tendencies to implement avoidance behaviours (last paragraph of page 3) making it unclear why any effect of COVID-related primes on imitation should be found in a more general population sample?

*Authors’ response: We have elaborated on our logic by reframing it within the context of situational primes (Pg. 3, Ln. 60-61). Indeed, it is relevant that the avoidance-type behaviours induced by potential infectious disease can also manifest following a mere situational prime as featured within the present study (Pg. 3, Ln. 50-54).*

*Moreover, aside from a theoretical perspective, it is worthwhile noting that the present study was motivated by a general interest in how the unprecedented scale of the current pandemic could influence human behaviour (Pg. 3, Ln. 41-46). In so doing, we could expand upon the catalogue of behaviours that have been known to be unexpectedly affected following the emergence of COVID-19.*

Method:

Reviewer’s comment/s: Please provide a clearer statement of the statistical power the sample size afforded. The two studies cited here are a decade old and more recent studies have used far larger sample sizes (again see Cracco et al. special issue / editorial). Although you can’t estimate the likely size of the effect you are trying to detect, you can at least state what effect you were powered to detect (e.g. ‘a sample of 20 participants per group provides 80% power to detect an effect of at least d=0.9 at an alpha level of .05’).

*Authors’ response: We have now incorporated a posteriori power analysis with a view to reporting the achieved statistical power (Pg. 10, Ln. 214-216). Please note, we have also retained the sensitivity analysis alluding to the minimum detectable effect size owing to a suggestion made by another reviewer.*

Reviewer’s comment/s: Please provide a figure illustrating the stimuli/task.

*Authors’ response: We have introduced a new figure that illustrates the transition across the protocol including the main stimulus-response task (Fig 1; Pg. 8, Ln. 166-169).*

Reviewer’s comment/s: 20 trials per condition is a lot fewer than in most automatic imitation tasks. Please provide rationale in terms of the number of trials needed to obtain a robust estimate of response time in each condition, and also indicate the mean/SD number of trials per condition remaining once false starts, delayed responses, and error trials were removed; and discuss how this relates to the number of trials required for estimating RT.

*Authors’ response: We concur with the argument of there being a low number of trials. However, it is relevant to consider the potential trade-off between ideally accumulating a large and more representative number of trials, and the pragmatics surrounding participant retention within a more remote participant-led data collection. We have attempted to clarify this point following the comments of a separate reviewer (Pg. 7-8, Ln. 160-162). At the same time, we do not know of any formal and statistically verified recommendation for the number of trials that are needed for this particular paradigm.*

*An initial coarse outlook of the data would seem to suggest that participants responded within a reasonable or typical range of reaction times (grand M = 548 ms), while we statistically replicated the classic automatic imitation effect (i.e., compatible < incompatible). However, in order to more precisely gauge whether the number of trials were adequately representative of the participants behaviour, we further inspected the reaction time data by calculating both the within-participant SD and median. Here, it is assumed that a more representative sample would produce a lower SD, while the mean and median reaction times would be somewhat similar to each other. Firstly, we found a mean within-participant SD = 120 ms (equating to a Coefficient of Variation (CoV) = 21%). Meanwhile, the below graphic indicates that the mean and median reaction times featured a highly similar trend. Thus, we infer that the low number of trials within the present study were still able to capture participants’ behaviour.*

*The fore mentioned outcomes were no doubt facilitated by the removal of trials that featured a false start, delay and/or error (Pg. 10, Ln. 216-217). It is important to clarify that this procedure led to the removal of very few trials as is indicated by the mean and SD of the number of trials (Pg. 10, Ln. 217-223; N.B., having stated the number of trials allocated to fast and slow time bins (see below response to comment), it is possible to aggregate them to infer the total number of trials removed).*

Results:

Reviewer’s comment/s: Given the very low number of trials I don’t think the bin analysis is appropriate. At the very least the authors should report the mean/SD number of trials remaining within each bin.

*Authors’ response: We have now reported on the mean and SD of the number of trials comprising the different time bins within each condition (Pg. 10, Ln. 217-223).*

It would be helpful to add the magnitude of the compatibility effects to Table 1 (i.e. the mean/SE of the Incompatible-Compatible difference across participants), please.

*Authors’ response: We have now incorporated a figure (Fig 2; Pg. 11, Ln. 243-246) that more appropriately reflects the compatibility effects in question.*

Last paragraph of page 9 – this states that the dependent variable was the compatibility effect ‘Further inspection of the compatibility effects as a function of movement time bins’ but then compatibility is reported as a factor in the analysis – so is this actually analysing RT not compatibility effect? Please clarify.

*Authors’ response: We have adapted the text to more explicitly communicate our meaning (Pg. 10, Ln. 230).*

First lines of page 10 – the Bayes’ factor associated with this interaction is in favour of the null hypothesis, suggesting limited support for different magnitude compatibility effects across the slow and fast bins – please check and then reflect this in your discussion (if indeed, given my points about trial numbers above, you retain the bin analysis).

*Authors’ response: We would like to thank the reviewer for this observation, and concur with their evaluation. As a result, we have more cautiously interpreted the reaction time bin analysis, and highlighted the failure of the Bayes Factor analysis to fully corroborate our suggestions (Pg. 15, Ln. 351). With this in mind, it is relevant to note that simultaneous frequentist and Bayesian statistical approaches do not always complement each other.*

Discussion:

Reviewer’s comment/s: Page 14 – ‘the present compatibility effects unfolded within the slow rather than fast reaction time bins’ – this isn’t quite accurate, the effects were greater in the slow bins but they were still present in the fast bins? A follow-up analysis of the simple effects of compatibility at each time bin would help to address this point.

*Authors’ response: We have amended the relevant text so that the reported compatibility effect was smaller, and not absent, within the fast time bins (Pg. 15, Ln. 349-351).*

Reviewer’s comment/s: I think the conclusion should be qualified ‘did not modulate automatic imitation in this group of young UK adults’.

*Authors’ response: We have qualified the concluding remarks as suggested (Pg. 16, Ln. 361-362).*
