# Supplementary material for: Does the threat of COVID-19 modulate automatic imitation?
Source: PLoS One. 2023 Apr 24;18(4):e0284936. doi: 10.1371/journal.pone.0284936 (PMC10124885; doi:10.1371/journal.pone.0284936)
Supplement: Attachment — Submitted filename: Response to Reviewers_R3_jr1.docx [file pone.0284936.s006.docx]

**Response to Reviewers**

**Please note, the references to page and line numbers are taken with respect to the manuscript version, ‘Revised Manuscript with Track Changes’.*

Reviewer #2: No response

Reviewer #3:

Reviewer comment/s: Please comment on the low number of trials per condition per time bin in the discussion.

*Authors’ response: We have now drawn attention to this pitfall including mention for future directions (Pg. 15, Ln. 349-353).*

Reviewer comment/s: Page 10 line 230: the dependent variable in this analysis is RT not incompatible-compatible difference, so this should say ‘further inspection of response times…’.

*Authors’ response: We thank the reviewer for this observation, and have subsequently altered the text (Pg. 10, Ln. 223).*

Reviewer comment/s: Page 11, line 238: does this t test refer to the compatibility effect in the fast or in the slow time bins? Please provide the equivalent test for the other time bin.

*Authors’ response: The t-test in question alludes to the compatible-incompatible reaction time difference for the slow compared to fast reaction time bins (Pg. 10, Ln. 229-231). Please note, while both bins indicated a compatibility effect (i.e., compatible < incompatible), the direct comparison between the slow and fast compatible-incompatible difference could more clearly highlight or decompose the purported Compatibility x Time Bin interaction (Pg. 10, Ln. 228-229).*
